# Does My Neck Make Me Clumsy? A Systematic Review of Clinical and Neurophysiological Studies in Humans

**DOI:** 10.3389/fpain.2021.756771

**Published:** 2021-10-11

**Authors:** Samantha C. Harman, Zhen Zheng, Julie C. Kendall, Dein Vindigni, Barbara I. Polus

**Affiliations:** ^1^School of Health and Biomedical Sciences, Royal Melbourne Institute of Technology University, Melbourne, VIC, Australia; ^2^School of Engineering, Royal Melbourne Institute of Technology University, Melbourne, VIC, Australia

**Keywords:** internal body schema, upper limb (UL), neck (MeSH), chronic neck pain, whiplash associated disorder (WAD), clumsiness, kinesthesia, proprioception (MeSH)

## Abstract

**Introduction:** Clumsiness has been described as a symptom associated with neck pain and injury. However, the actuality of this symptom in clinical practice is unclear. The aim of this investigation was to collect definitions and frequency of reports of clumsiness in clinical studies of neck pain/injury, identify objective measures of clumsiness and investigate the association between the neck and objective measures of clumsiness.

**Methods:** Six electronic databases were systematically searched, records identified and assessed including a risk of bias. Heterogeneity in designs of studies prevented pooling of data, so qualitative analysis was undertaken.

**Results:** Eighteen studies were retrieved and assessed; the overall quality of evidence was moderate to high. Eight were prospective cross-sectional studies comparing upper limb sensorimotor task performance and ten were case series involving a healthy cohort only. Clumsiness was defined as a deficit in coordination or impairment of upper limb kinesthesia. All but one of 18 studies found a deterioration in performing upper limb kinesthetic tasks including a healthy cohort where participants were exposed to a natural neck intervention that required the neck to function toward extreme limits.

**Conclusion:** Alterations in neck sensory input occurring as a result of requiring the neck to operate near the end of its functional range in healthy people and in patients with neck pain/injury are associated with reductions in acuity of upper limb kinesthetic sense and deterioration in sensorimotor performance. Understanding the association between the neck and decreased accuracy of upper limb kinesthetic tasks provide pathways for treatment and rehabilitation strategies in managing clumsiness.

## Introduction

Neck pain is common (1), and moderately to severely limits activity in 17–19% of the population (2). Complications from neck pain and injury, including Whiplash Associated Disorders (WAD), are frequently reported in the literature. These include the development of chronic pain (3), dizziness (often referred to as cervical vertigo) (4, 5) and disturbances in balance (6). Bring and Westman (7) made the first clinical reference to the symptom of “fumbling” as a late symptom of those suffering from traumatic neck pain. They noted that this symptom was usually described as a tendency to drop things or an insecurity or difficulty in gripping. As recognized by Bring and Westman (7) the clinical picture presented by traumatic neck pain patients is complex yet objective findings are weak. Later, Treleaven et al. (5) reported that 30% of whiplash patients described feeling “clumsy” as a symptom, exacerbating feature, or concurrent symptom associated with their dizziness or unsteadiness. The authors also found that these patients had greater cervical joint position errors when returning their head to its natural head posture after actively extending or rotating their head. These larger errors, most frequently an overshoot in estimating the position of their natural head position, were attributed to deficits in the proprioceptive information available from agonist neck muscles.

Subsequent reports referred to disturbances in sensorimotor control as a likely consequence of damage to neck structures as a result of neck injury (8–10) and included symptoms such as deficits in coordination of upper limb movement (11) fumbling (7, 12) or clumsiness (12, 13).

Knox et al. (13) reported that rotation of the neck just prior to the point of pain reproduction, was associated with an increased elbow joint position error in a clinical population of people with whiplash injury. A further study reported that decreased elbow joint position sense accuracy occurred even in healthy people at end range of neck rotation (14). The authors of this latter study (14) suggested that the processing of neck proprioceptive information at the extreme of neck range of motion (ROM) might be responsible for the decreased acuity of upper limb position sense. The result of the Knox et al. study (13) that demonstrated increased elbow joint position error when the head-neck was rotated to a point just before participants reported an increase in neck pain or discomfort was interpreted as those suffering neck pain nearing the end point of their functional neck range of motion. The results of the Knox et al. study therefore may be considered to mirror the results of the Knox and Hodges study (14).

These studies point to the role that the neck plays in the brain's understanding of where its body parts are positioned in relation to each other. The central nervous system must be able to differentiate between: the whole body moving; the body changing position relative to the head; and movement of just the head. Roll et al. (15) described the contribution of whole-body proprioceptive inputs, including those from the neck, in the construction of an internal representation of body segments in relation to each other and in extrapersonal space. Therefore, proprioceptive signals from the neck play a pivotal role in both the construction and continual updating of the CNS' internal body representation. This requires the integration of vestibular, neck and trunk proprioceptive signals and of these, the neck proprioceptive signals provide critical information regarding head-neck information relative to the trunk (16).

So, the question that arises is, what evidence do we have that neck pain is associated with the symptom of clumsiness? As noted above Bring and Westman (7) made the first clinical reference to “fumbling” which was then given an operational definition by Sandlund et al. (11) and Knox et al. (13) the latter who described fumbling or clumsiness as a deficit in coordination of the upper limb. Therefore, the purpose of this systematic review was to review evidence of an association between the neck and clumsiness, when clumsiness was defined as upper limb position and movement sense and sensorimotor task performance. This was investigated in both neck pain and/or injury and healthy cohorts. In particular, the aims were to:

1. Summarize frequency of reports of clumsiness from the clinical literature where clumsiness is associated with neck pain and/or injury and how clumsiness was defined in this context;
2. Review how clumsiness, a deterioration in performance of an upper limb sensorimotor task as this symptom is operationally defined from clinical studies, is investigated in cohorts experiencing neck pain and/or injury; and in a healthy cohort where studies used upper limb sensorimotor task performance as the outcome and non-artificial neck exposures; and
3. Review the evidence that there is an impairment in performance of upper limb sensorimotor tasks in the presence of neck pain or injury or, in a healthy group when a natural intervention is applied.

## Methods

This review was carried out in line with Preferred Reporting Items for Systematic Reviews and Meta-Analyses (PRISMA) statement (17). The PRISMA 2009 27-item checklist has been provided as Supplementary Material A. A review protocol was established and included search strategies, inclusion and exclusion criteria and methods of analysis. An outline of this protocol has been listed below. A full copy of the protocol is available upon request.

### Selection Criteria

To be included, the studies must have met all the selection criteria.

#### Type of Participants

1. human and between the ages of 18–65 years;
2. acute, sub-acute or chronic non-specific neck pain and/or neck injury and/or whiplash;
3. and/or healthy participants where as part of the testing protocol there was either an induced change in the neck position of the participant or fatigue of their neck muscles;
4. excluded if participants had a history of cancer, fracture, infections, rheumatological disorders, neurological disorders, or surgical procedures for spinal or extremity disorders.
5. excluded if there was insufficient documentation or information on participant demographics or if data extraction was not possible.

#### Type of Study Design

The type of study designs included case control, cohort and randomized control trials. Articles were included if they were published as a full paper or an abstract with sufficient detail to extract the main attributes of the study.

Studies were limited to those published in peer-reviewed journals, without language restriction. Publications were excluded if they were duplicate studies or reviews.

#### Types of Outcome Measures

To be included, studies had to report one of the following outcome measures: upper limb joint position sense; upper limb joint position error; upper limb movement task; clumsiness and/or fumbling; upper limb proprioception; or coordination in the extremity/limb. Results were excluded from analyses if methods involved the use of artificial stimulation of senses (such as galvanic vestibular stimulation or vibration) or microgravity as these were considered beyond the scope of natural interventions.

In the initial design of this review the lower limb was included in the search terms. No papers pertinent to lower limb and clumsiness were found, therefore this review only reported on clumsiness associated with the upper limb.

### Data Sources and Search Strategy

Searches (initial and updated) were conducted using PubMed, EMBASE, CINAHL, Index to Chiropractic Literature, Cochrane Library and Scopus from date of inception to 3/3/21. This was conducted by the first author and checked by another author (JK).

A comprehensive search strategy was developed by identifying and listing all potentially relevant search terms, categorizing these into specific search phrases and combining them using Boolean terms. Terms included keywords and phrases “neck pain,” “neck injury,” “whiplash,” “healthy,” “position sense,” “kinesthesis,” “clumsiness,” “proprioception,” “joint position sense,” “upper extremity.” PubMed was searched using MeSH terms. A sample of the PubMed search strategy is provided in Supplementary Material B. The detailed search strategy is available upon contacting the corresponding author.

The reference lists of selected articles retrieved in the original online search were also screened for relevant studies not identified through electronic searches by the lead and last author. Citation searches of the identified relevant studies were conducted using PubMed and Scopus databases.

The first and last author (SH and BP) screened the title and abstracts of the articles based on the inclusion/exclusion criteria and full reports were obtained of all the studies identified as potentially eligible. If any title or abstract did not provide enough information to decide whether the inclusion criteria were met, then the full text was obtained. All of the full-text studies were then independently evaluated. Discrepancies in judgement were first resolved by discussion with two reviewers, however, if consensus was not reached, a third reviewer was used to arrive at a decision.

### Risk of Bias

An assessment of the risk of bias and precision was conducted by the lead and last author using the risk of bias assessment tool of Viswanathan and Berkman (18). As reported by these authors the tool has been developed to evaluate “…the degree to which the effects reported by the study represent the ‘true' causal relationship between exposure and outcome.” This assessment tool has an item bank with a series of items applicable for observational studies from which relevant items may be selected and defined in order to assess their internal validity. Individual items from this bank identified by the authors as relevant to this review were then selected, defined and applied to each of the studies retrieved for analysis, with the subsequent risk of bias findings tabulated (see Table 1). To support this quality assessment, a numerical value was assigned to each criterion. If an assessment was determined as high, it received a numerical score of 1. Any lower assessment received a score of 0. We then classified the overall score of quality as low (0–2), moderate (3, 4) or high (5, 6) after Zhang et al. (19). The sum of all values provided the basis to quantitatively assess overall quality.

**Table 1:** Risk of bias [adapted from Viswanathan and Berkman (18)].

**References**	**Study** **design**	**Are critical** **inclusion/exclusion** **criteria clearly** **stated?**	**Are the** **inclusion/exclusion** **criteria measured** **using objective** **measures?**	**What is the** **level of detail** **in describing** **the intervention** **or exposure?**	**Are outcomes** **assessed using** **objective measures,** **implemented consistently** **across all** **study participants?**	**Are the statistical** **methods used to** **assess the primary** **benefit outcomes** **appropriate to** **the data?**	**Are results** **believable taking** **study limitations** **into consideration?**	**Quality score** **(out of 6)**
Haavik and Murphy (20)	P	Y	N	H	Y	N	P	3
Huysmans et al. (21)	P	Y	Y	H	Y	Y	Y	6
Knox et al. (13)	P	Y	Y	H	Y	Y	Y	6
Knox and Hodges (14)	P	Y	Y	H	Y	Y	Y	6
Sandlund et al. (11)	P	Y	Y	H	Y	Y	Y	6
Sandlund et al. (22)	P	Y	Y	H	Y	Y	Y	6
Guerraz et al. (25)	P	N	U	H	Y	Y	Y	4
Berger et al. (26)	P	N	N	H	Y	Y	Y	3
Fookson et al. (28)	P	N	N	H	Y	Y	Y	3
Rossetti et al. (33)	P	N	N	H	Y	Y	Y	4
Blouin et al. (27)	P	N	N	H	Y	Y	Y	4
Guerraz et al. (29)	P	Y	N	H	Y	Y	Y	5
Zabihhosseinian et al. (30)	P	Y	Y	H	Y	Y	Y	6
Guerraz et al. (32)	P	Y	Y	H	Y	Y	Y	6
Zabihhosseinian et al. (31)	P	Y	Y	H	Y	Y	Y	6
Sittikraipong et al. (23)	P	Y	Y	H	Y	Y	Y	6
Steinmetz and Jull (24)	P	Y	Y	H	Y	Y	Y	6
See and Treleaven (12)	P	Y	Y	H	Y	Y	Y	6

*P, Prospective; Y, Yes; N, No; U, Can't Determine, measurement approach not reported; H, High; M, Medium; P, Partially; N/A, Not Applicable*.

### Data Extraction

General information regarding participants' characteristics and demographics (see Table 2) was obtained by the lead and last author, including the number of participants, age, gender, criteria for inclusion/exclusion, health status (i.e., healthy, idiopathic neck pain, whiplash), questionnaires used to measure pain and/or disability, function and mental status, and pain duration and intensity. In order to standardize the data extraction process between the reviewers, detailed data extraction sheets were devised and used to acquire information concerned with research questions about the role of the neck in joint position and movement sense and motor performance of the upper extremity. These results are shown in Tables 3–5. In particular information was sought to determine (i) whether clumsiness was mentioned and how it was operationally defined, (ii) if clumsiness was not mentioned, an explanation for why joint position sense and/or motor performance were used as outcome measures, (iii) the association between neck position or movement and joint position sense or motor performance of the upper limb, (iv) the association between neck pain/injury and joint position sense or motor performance of the upper limb, (v) previous pain intensity and duration, (vi) neck and body position of participants during the tests, (vii) visual condition (eyes open/closed) and (viii) the method used to measure joint position sense/motor performance (e.g., electrogoniometer, electromagnetic tracking).

**Table 2:** Participant demographics.

**References**	**Inclusion and** **exclusion criteria**	**Neck pain** **injury; number/** **male/** **female; and** **Mean age** **years (range)** **and (SD)**	**Healthy; number/** **male/** **female; and** **Mean age** **years (range)** **and (SD)**	**Questionnaires for** **pain, disability,** **function and** **mental status**	**Pain: duration** **Mean (SD)** **and Intensity** **(range)**
Haavik and Murphy (20)	Inclusion- Healthy or SCNP. SCNP defined as recurring neck dysfunction e.g., mild neck pain, ache, ± stiffness ± history of known neck trauma; not constantly symptomatic and no treatment of their neck complaint yet sought. Exclusion- History shoulder/elbow pain, current pain anywhere in the body, diagnosed degenerative joint disease, any medical condition affecting the sensory system; previous treatment for neck pain. Contraindications to cervical spine manipulation such as previous fractures, high blood pressure and metabolic, inflammatory, or neoplastic disease.	SCNP; 25/M15/F10 Mean 25.7 ± 4.3	18/M5/W13 Mean 23.2 ± 9.5	None reported	Duration not reported; No acute episode on day of testing
Huysmans et al. (21)	Inclusion- Pain right neck and upper extremity for at least 4 weeks in last 3 months, 4 days in the last week and on the day of measurement, all considered pain work related and worked for at least 4 h/day on computer; all right hand dominant. Exclusion- Specific (neurological) pathology, acute trauma, injury or birth defect that could have caused their pain, no prescribed medication.	Pain in neck and upper extremity; 23/M4/F19 Mean 43.0, (SD 10.7), (range 24–61)	26/M4/F22 Mean 42.4, (SD 11.1), (range 24–62)	11-point numerical scale ranging from 0 no pain to 10 worst pain. Dutch version 30-item Disabilities of the arm, shoulder and hand questionnaire	3.7 yrs (SD 2.8). Worst pain last 3 months 6 (3–10) Average pain last 3 months 4 (2–9) Pain day of measurement 4 (1–10)
Knox et al. (13)	Inclusion- Chronic Whiplash II Group (sustained more than 3 months ago) and Healthy Group Exclusion- Sustained a head injury, loss of consciousness, upper limb nerve injury as a result of their whiplash accident. Also vestibular pathology, or experienced right arm pain since their whiplash injury. Chronic WAD group, all right-handed.	Chronic Whiplash; (type II) 9/2M/7F Mean 30 ± 9	11/5F/6M Mean 26 ± 3	NDI, Speilberger State-Trait Anxiety Questionnaire (STAI)	>3 months; Time since accident 22 (4–46) months; >30/100 (NDI). Baseline pain 2.0 (0–3.5) cm; Change in pain 0.9 (−0.1 to 2.2) cm
Knox and Hodges (14)	Inclusion- Healthy Exclusion- History neck, shoulder, elbow pain, current pain in another body region, diagnosed degenerative joint disease, or any medical condition affecting the sensory systems.	None reported	10; Mean 29 ± 5	None reported	None reported
Sandlund et al. (11)	Inclusion- Chronic Whiplash WAD II & III (whiplash trauma > 6 months ago); and Healthy. Both groups RH. Exclusion- No recent injuries to right arm or shoulder fractures, joint sprains or luxations <2 years ago, conditions of neurological disease, diabetes or fibromyalgia. Healthy Group no history head, neck, shoulder trauma, no current shoulder, arm problems or longer periods of constant or intermittent neck-shoulder pain. Both groups no fractures or diagnosed rhizopathia.	Chronic whiplash (type II and III) 37/17M/20 F Mean 39.9 (SD 9.7)	41/15M/26F Mean 39.0 (SD 9.6)	VAS pain, Pain Disability Index, 20 item Functional Self-Efficacy Scale, Short Form Health Survey SF-36	Minimum 6 months: 6 months to 13 years (median 2.5). Intensity not reported.
Sandlund et al. (22)	Inclusion- Neck pain at least 3 months duration and score >10 Neck Disability Index (NDI). WAD group onset symptoms accident related & occurred within 2 weeks of accident. All RH, 20–50 years old & able to perform voluntary arm movements with arm elevations above 110° and > 25° axial rotation of head. Exclusion- Surgery of neck, shoulder, or back, injuries with fractures or luxations to the neck or shoulders, conditions of neurological or rheumatic disease or fibromyalgia. Control group no history head/neck/shoulder trauma and no current neck/shoulder pain or longer periods of constant or intermittent neck-shoulder pain.	NS; 24/10M/14F;WAD; 21/10M/11F;MeanNS 37 ± 9WAD 36 ± 5	22/9M/13F Mean 37 ± 10	NDI, Short Form Health Survey - 36, VAS pain, Swedish validated version 27 Disability of the arm, shoulder and hand (DASH), TAMPA scale of Kinesiophobia (TSK), Self-efficacy Scale and Additional questions not covered by other questionnaires.	NS 60 [12-368] weeks; WAD 73 [22-215] weeks; VAS NS 47 (±23); VAS WAD 60 (±22)
Guerraz et al. (25)	Inclusion- Healthy Exclusion- No relevant medical history. All RH	None reported	Exp 1: 9/9 M (22–33) Exp 3: 6/6 M (22-36)	None reported	None reported
Berger et al. (26)	Inclusion- Healthy All RH	None reported	14 (20–28).	None reported	None reported
Fookson et al. (28)	All RH	None reported	6/3M/3F; (35–60)	None reported	None reported
Rossetti et al. (33)	Inclusion- Healthy, RH, visual acuity equal to at least 21 cycles/deg.	None reported	6/4M/2F; (22–45)	None reported	None reported
Blouin et al. (27)	Inclusion- Healthy, RH	None reported	9; Mean 27.2 (22–32)	None reported	None reported
Guerraz et al. (29)	Inclusion- Healthy, RH	None reported	12/9M/3F; Mean 23.2 (19–27) (SD 2.5)	None reported	None reported
Zabihhosseinian et al. (30)	Inclusion- Healthy, RH	None reported	12/6M/6F; Mean 21.7 ± 3.6	None reported	None reported
Guerraz et al. (32)	Inclusion – Healthy, RH Exclusion - no history of vestibular, visual, or neuromuscular disease	None reported	Exp 1: 12/7M/5F; Mean 24.3 (19-32) (SD 3.5) Exp 2: 7/3M/4F Mean 26 (19-40) (SD 8.5)	None reported	None reported
Zabihhosseinian et al. (31)	Inclusion – Healthy, RH, absence of neck pain	None reported	Fatigue group 12/6M/6F; Mean 20.5 (SD 2.1); Control group 12/&M/5F 20.76 (SD 0.9)	NDI	None reported
Sittikraipong et al. (23)	Inclusion – NS =/> 3 months; NDI (Thai version) =/> 10/100 Asymptomatic controls age and gender matched; no history of neck pain & dizziness for past year. Exclusion criteria- history of trauma/surgery to head/neck upper back and lower extremities, neurological disorders, uncorrected visual problems, suspected vestibular pathology, use of medications that could influence reaction and response times and/or eye coordination.	60 chronic NP Age 33.6 ± 10.590% F	60 controls Age 31.2 ± 10.5 95% F	NDI; VAS; for NP group only	>3 months; ≥10/100 on NDI
Steinmetz and Jull (24)	Inclusion - Violin and viola players (violinists) > 18 years, play their instruments between 15–20 h per week. Violinists who reported neck pain associated with playing their instrument included in symptomatic group. Asymptomatic group included violinists with no report of neck-shoulder pain for past 12 weeks and not reported regular pain episodes with playing. A further control group of non-playing age and gender matched university volunteers recruited as a true comparison Exclusion criteria - history of trauma/ surgery to the neck, shoulder, or arm regions and any history of neurologic or chronic orthopedic/rheumatologic diseases.	22 violinists with NP, 17F/5 MAge 27.6 ± 10.8	21 violinists 15F/6M Age 28.2 ± 11.9 21 healthy non-musicians 15F/6M Age 31.8 ±9.8	Demographic data (age, sex BMI)For musicians – time of playing instrument per day/per week and how many yearsNDIMusicians with neck pain - VAS	No measure of duration VAS 5.0 (±2.0)
See and Treleaven (12)	Phase 2 only Inclusion - Persistent symptoms of at least 3 months; Minimum NDI score of 10% Exclusion - history of cervical or upper limb fractures or dislocations, neurological disorders, previously diagnosed central nervous system diseases or inability to comprehend the task Asymptomatic group inclusion: no history of head, neck or upper limb trauma and no current neck or upper limb problems. Participants were excluded if they had recent (<2 years) injury to their upper limb and known neurological disease, fibromyalgia or articular diseases affecting the cervical, shoulder, elbow, wrist or hand.	6 participants with persistent WAD (18-70 years) Female 87.5%	13 age and gender matched asymptomatic volunteers	NDI; Patient specific functional scale (average score out of 10 by totalling each activity/3); Disability of the arm, shoulder and hand (DASH/100), VAS pain	Neck upper limb pain ≥ 3 months, VAS pain intensity 50.60 (±20.1)

*F, Female; M, Male; Exp, Experiment; RH, Right Handed; LH, Left Handed; WAD, Whiplash Associated Disorder; NS, Non-specific neck pain; NDI, Neck Disability Index; JPE, Joint Position Error; BP, Baseline Pain; SCNP, Subclinical Neck Pain; cm, centimeters; RH, right handed*.

**Table 3:** Definitions of clumsiness and investigations of performance of upper limb kinesthestic tasks.

**References**	**Is clumsiness defined** **and if so how** **is it operationally defined?**	**Why were upper limb kinaesthetic tasks employed?**
Haavik and Murphy (20)	No	Investigation of change in head-neck position on an elbow JPS accuracy task in participants with subclinical neck pain and healthy controls
Huysmans et al. (21)	No	Investigation of upper limb position sense acuity and tracking performance, pen pressure and muscle activity in a tracking task in participants with neck pain and healthy controls
Knox et al. (13)	Yes. Deficits in coordination of upper limb movement	Investigation of change in head-neck position in accuracy of an elbow JPS task in WAD and healthy controls
Knox and Hodges (14)	No	Investigation of change in head-neck position to end point of participant's range of motion on accuracy of reproducing a previously presented target angle of elbow flexion in healthy participants
Sandlund et al. (11)	Yes. Impairment of shoulder proprioception	Investigation of ipsilateral shoulder position-matching task in patients suffering WAD and self-rated function and pain ratings and healthy participants
Sandlund et al. (22)	No	Investigation of goal-directed arm movement to a visual target in participants with chronic non-traumatic non-specific neck pain, WAD and healthy controls.
Guerraz et al. (25)	No	Investigation of change in head-trunk relation on accuracy in reproducing a geometric drawing in healthy participants.
Berger et al. (26)	No	Investigation of changing head-to trunk positions on the accuracy of arm pointing task in cosmonauts and healthy population.
Fookson et al. (28)	No	Investigation of changing position of the head relative to the body in upper limb pointing accuracy task.
Rossetti et al. (33)	No	Investigation of relationship between accuracy of visual localization and eye and head positions. In part it examined effect of different head positions on pointing accuracy task in healthy participants.
Blouin et al. (27)	No	In part investigated head-trunk rotations with stationary head fixed and with neck-muscles relaxed and near maximal neck-muscle activity without any head motion, on pointing accuracy in healthy participants.
Guerraz et al. (29)	No	Investigation of prolonged head-neck tilt (return phenomenon) on accuracy in reproducing a geometric drawing in healthy participants
Zabihhosseinian et al. (30)	No	Investigation of dorsal neck muscle fatigue on accuracy of an upper limb (elbow) joint repositioning task in healthy participants.
Guerraz et al. (32)	No	Investigation of ability to reproduce a geometric shape after head tilt either from memory or visually guided, without vision of the upper limb.
Zabihhosseinian et al. (31)	No	Investigation of dorsal neck muscle fatigue on eye-hand tracking accuracy in healthy participants
Sittikraipong et al. (23)	No	Hypothesis – individuals with neck pain will have slower reaction & response times and impaired hand-eye coordination compared to asymptomatic controls; also a relationship between reaction and response times and hand-eye coordination and clinical features of neck pain
Steinmetz and Jull (24)	No	Hypothesis: 1. violinists with neck pain will report increased sensitivity to hot/cold stimuli and pressure (hyperalgesia) and 2. Violinists with pain will exhibit altered arm and hand sensorimotor performance
See and Treleaven (12)	Yes. Upper limb functional difficulties	Aim: to perform a series of case studies on patients with persistent WAD to investigate whether there is evidence for poorer motor performance on the BEP1 in WAD compared to age and gender matched asymptomatic individuals and if so, whether this relates to reported functional upper limb complaints in persistent WAD

**Table 4:** Association between the neck and changes in accuracy in completion of upper limb sensorimotor tasks.

**References**	**Head-neck** **movement performed**	**Body position**	**Upper limb** **kinesthetic task**	**Dominant upper** **limb used in** **kinesthetic task**	**Instruments used for** **measurement of upper** **limb performance**	**Outcome measures reported** **after head-neck movements,** **exposures or interventions**	**Results**
Haavik and Murphy (20)	Neutral, left rotation, flexion, combined flexion and left rotation	Supine	Elbow joint position tested in mid-range of joint movement after experimenter passively moved elbow to a target angle; eyes closed	Partially	Electrogoniometer to measure joint position angle	Absolute error, constant error and variable error between presented angle and reproduced angle for each head-neck position recorded pre and post cervical manipulative thrust	Control group better at reproducing target angle than SCNP group at baseline. Accuracy of reproducing target angle in SCNP group improved in neutral and left rotation post intervention.
Huysmans et al. (21)	Straight ahead	Standing – position sense acuity; sitting – tracking task	Joint position sense acuity task; participant able to visualize location of target on top of the tablet; participant located position of the target under the tablet which was obscured from vision; tracking a target dot that moved quasi-randomly across a computer screen; applied pen pressure during tracking task;	Yes	Position sense acuity task – digitizing tablet with marked target points. Tracking task- participant tracked a target dot that moved randomly across the screen using a pen on a digitized tablet. Axial pen pressure; upper limb / neck muscle activation via sEMG during performance of tracking task	Variable error for position sense acuity; tracking performance assessed by % time on target, mean distance between center of target and center of cursor; S.D. between distance from center of target and center of cursor; percentage lag between position of cursor and moving target	Position sense task performance impaired compared to controls; pain group had reduced tracking performance; no difference in pen pressure or muscle activation between groups.
Knox et al. (13)	Head-neck movement limited to ROM that did not increase WAD participant's pain. Head positions: neutral (control), flexion, left & right rotation. Range 20**°** to 50**°** for WAD; Control 30**°**.	Supine	Elbow JPS tested in mid-range of joint movement after experimenter passively moved arm to target angle. Eyes closed throughout trial.	Partially	Electrogoniometer to measure joint position angle	Accuracy of elbow angle for each head position between target and reproduced elbow angle was assessed using absolute error, constant error and variable error	Changes in head-neck position increased absolute error of JPE in WAD group but not control with smaller angles of neck rotation in WAD compared to healthy
Knox and Hodges (14)	Neutral, left rotation, flexion, combined flexion and left rotation	Supine	Elbow JPS tested in mid-range of joint movement after experimenter passively moved arm to target angle. Eyes closed throughout trial.	Unknown	Electrogoniometer to measure joint position angle	Accuracy of elbow angle for each head position between target and reproduced elbow angle was assessed using absolute error, constant error and variable error	Absolute and variable joint position errors greater when target angle reproduced with neck in flexion, rotation and combined flexion/rotation compared to head in neutral.
Sandlund et al. (11)	Head straight	Seated	Shoulder joint repositioning task; a blindfold was worn.	Yes	Electromagnetic tracking system	Variable error of the difference in position of shoulder target angle and reproduced target angle. Association between proprioceptive acuity and questionnaires including disability, functional self efficacy scale, QoL evaluation (short form health survey) and VAS scores	WAD group showed significantly lower acuity in reproducing target compared to healthy controls (*P* = 0.003); moderate correlation between low self-rated physical functioning and low proprioceptive acuity (*P* = 0.042)
Sandlund et al. (22)	Head straight	seated	Performance of fast and accurate arm pointing movements to a visual target; eyes open	Yes	Electromagnetic tracking system	Variable error of end-point acuity measured as difference in position of pointer located at the tip of the hand and position of target measured via global coordinate system; speed of movement	Significant difference in end point acuity in goal-directed reaching between control and non-specific neck pain for both depth (*P* = 0.03) and vertical (*P* = 0.032), control and WAD in depth direction (P=0.010)
Guerraz et al. (25)	Experiment 1. Head aligned with trunk or tilted toward left/right shoulder 2. Head aligned with trunk 3. Head tilt	Experiments 1 and 2: Seated + GVS stimulation Experiment 3 Supine	Drawing task – geometric shapes, square and diamond; eyes open while reproducing the geometric figure; eyes then closed, head tilted and drawing task repeated	Yes	Electromagnetic tracking device (Polhemus 3 space Fastrak)	Orientation of 4 segments of each geometric shape were analyzed using regression analysis and then transformed into angular measures	Experiment 1 – drawings rotated in the opposite direction to tilt. (*P* < 0.01) Experiment 2 – significant deviation of drawings toward anode (*P* < 0.05); Experiment 3 – supine and seated, significant rotation of drawings; seated and supine (*P* < 0.01); supine – greater variability in in shape reproduction compared to seated (*P* < 0.05)
Berger et al. (26)	Head straight; head rotated to right/left shoulder, head tilted to right/left shoulder	Seated	Aimed arm movements pointing toward 2 visual targets 11.7 degrees left and right in front of the participant. Targets were 2 flashing LEDs and participants pointed to target 6 times as accurately as possible with eyes open. Eyes were then either open/closed, repeated the learned movement from memory in different head positions.	Yes	Position of arm monitored MONIMER 3D registration system (3 IT-LED's and 2 IR scanning cameras)	Length and duration of arm movement, slant of arm movement; curvature of arm movement	Slant of movement plane of arm greater in neck rotation compared with lateral flexion. Comparison of movement of head to right and left – slant, amplitude and duration (*P* < 0.001) and difference in horizontal offset and curvature (*P* < 0.01). Movement toward direction of head position generates larger slant compared to movement opposite head position.
Fookson et al. (28)	Straight ahead, extreme head rotation right and left after presentation of target angle. After target presentation, eyes were closed for rest of trial. 4/6 participants completed additional experiment – trunk rotated 70–75 degrees counterclockwise, head turned to right throughout experiment	Seated	Participants faced presented targets in 3D space presented in random order in 5 locations in 2 planes in space. Each target presented for 1.5 secs. Participants then closed their eyes. One second later participants then touched the remembered target location	Yes	Infra-red emitting diodes and IR scanning cameras to record arm movement and robotic arm movement	Pointing errors computed in a spherical should-centered coordinate system – origin initial shoulder position of each movement. Azimuth elevation and radial distance errors were calculated. Constant errors used to measure pointing accuracy	Final position of the arm systematically shifted in direction opposite to that of extreme head rotation. Azimuth error significantly different for head-turn right to control (*P* < 0.05); head turned left to control (*P* < 0.01); head-turned right vs. head turned left (*P* < 0.001)
Rossetti et al. (33)	Head positions 0, 40, 80 degrees to right	Seated	Accuracy of target pointing – without vision of moving hand.	Yes	Infrared camera to record finger movement via invisible infrared-emitting diode attached to fingertip	X and y coordinates of end point of finger movement computed spatial errors - constant radial, angular surface errors. Regression between pointing error and 3 head angle computed.	Scatter (variability) of surface error increased with increasing head rotation (*P* < 0.05)
Blouin et al. (27)	Trunk rotation against fixed head with neck muscles relaxed (relaxed neck condition); Trunk rotation + neck muscle contraction (activated-neck condition); Whole body + head rotation (vestibular condition)	Seated	Pointing, using unseen index finger, at a previously memorized visual target	Yes	Electromagnetic tracking of final finger position	Mean perceived target position of all trials for each condition (cervical; neck muscle contraction and vestibular); pointing variability (estimate of reliability of performance)	No significant effect of condition on mean final pointing position (*P* > 0.05). Significant effect of experimental condition on variability (*P* < 0.0001). Vestibular condition associated with increased variability; no difference between neck conditions. Interpretation – neck more accurate than vestibular; use of a body-centered (target-trunk) reference system
Guerraz et al. (29)	Head straight or passively tilted toward right or left shoulder and maintained in tilted position for 15 min (return phenomenon)	Supine	Draw 4 X 25-30 cm straight lines in alignment with the trunk; first toward navel, then return to original starting point; blindfolds were worn	Yes	Electromagnetic tracking with sensor attached to finger tip	Line orientation defined as angular deviation of the drawn line	Angular deviation significantly affected by head orientation (*P* < 0.01). Orientation deviated in opposite direction to head tilt.
Zabihhosseinian et al. (30)	Induced neck muscle sub-maximal fatigue with head in neutral position	Standing	Recreation of a previously presented elbow joint angle; eyes closed	Yes	3D motion capture system with IR markers positioned on upper arm and wrist; elbow joint angles calculated as change in wrist position to change in upper arm position during joint motion	Absolute, constant and variable error of the difference between presented target angle and reproduction of the angle.	Significant effect of muscle fatigue on absolute error (*P* < 0.0001). No change in constant or variable error
Guerraz et al. (32)	Head either aligned with the trunk or tilted 30 degrees toward left or right shoulder	Supine	Reproduction of a mirror-reflected geometric figure using the right index finger without seeing their hand. The shape was either observed during the task or eyes were closed. Arm was outstretched & flexed to 90 degrees so that finger touched the board which was orientated in the frontal plane. Wrist and index finger were secured to prevent movement and minimize motor strategies adopted by different participants.	Yes	Finger displacements recorded in 3D using Polhemus Fastrak (magnetic sensor)	Extrinsic and intrinsic characteristics of each individual figure reproduction using (a) mean segment orientation of geometric figure (extrinsic) (b) shape of figure (angular deformation index and (c) length of segments (size reproduction; intrinsic)	With visual supervision and memory conditions deviations in reproduction of shape in direction opposite head tilt. Deviations less with vision and head tilted left. Perceptual visual bias induced by head tilt also evaluated. Participants asked to align figure with their median trunk axis. Figure drawing was perceived parallel with the trunk when it actually tilted in the direction of the head.
Zabihhosseinian et al. (31)	Cervical extensor muscle (CEM) fatigue vs. control	Seated	Participants used index finger to position a circular object at the center of a square target using a fully extended arm visualized on a screen with (a) vision of the target (b) target hidden. Position of square target was constant. Position of circular object was randomized to ensure unpredictability throughout eye-hand tracking task. In hidden target condition, square was not visible. Both conditions, dragging the circular object to target was visible.	Yes	Task error measured in pixels – how far away Centre of circle was away from Centre of square.	Measurement of angle of path deviation and end-point position for controls and for muscle fatigue group – pre and post fatigue	significant differences between the target with vision and the hidden condition for both groups between pre- and post-fatigue trials in angle of trajectory (*p* = 0.0001), and distance from release point to the target (*p* = 0.0001). Significant differences occurred in the hidden target condition for the fatigue group immediately post fatigue (*p* = 0.018) for distance from release to the target.
Sittikraipong et al. (23)	Not stated	Not stated	1a. Reaction time: participants asked to press and release right mouse button as fast as possible when left mouse button illuminated 1b. Participant asked to press right mouse button when they saw light illuminated. 2. Participants required to trace shapes as fast as possible	Yes	1a. Reaction and response time: hand-held electronic timer with modified computer mouse; light stimulus on left mouse button & finger depression response switch on right mouse button; 1b. Response time: modified computer mouse positioned 40 cm from participant's starting hand position 2. Hand-eye coordination task: participants held stylus with iPad set flat on a table. Participants required to trace shapes of increasing complexity	Differences in reaction and response times between groups; For hand-eye coordination tasks: differences in time taken and errors made. Spearmans's correlation coefficient used to explore association between reaction and response time, hand-eye coordination and clinical features of neck pain (NDI-TH, VAS, duration of neck pain)	Reaction and response times significantly slower for pain groups compared to control (*p* < 0.001); hand-eye coordination task – neck pain group took longer to trace shape at most difficult level (*p* = 0.03). Neck disability scores correlated with hand reaction time (*r* = 0.4, *p* = 0.005) and time taken in hand-eye coordination tasks (*r* = 0.2 for all levels of difficulty, *p* < 0.05). Reaction and response times correlated with time taken in hand-eye coordination task (*r* = 0.2–0.4, *p* < 0.01)
Steinmetz and Jull (24)	Not stated	Not stated	1. Reaction time 2. Speed of movement; 3. Accuracy of movement 4. Movement coordination	Not stated	Human performance Measurement/Basic Elements of Performance (HPM/BEP) used to measure motor aspects of upper limb/hand	Reaction time – time delay between a light stimulus and release of hand from a central touch plate to a target plate in 3 tasks (a) simple reaction time, (b) 2 choice reaction times. Speed of movement calculated by dividing distance between central and target plate by time taken for hand movement; Accuracy – participants tapped 2 narrow plates alternately with their index finger as fast & accurately as possible for 10 s; Coordination – calculated as combination of % correct hits and average speed of movement	No significant difference between symptomatic and non-symptomatic musicians and non-musician group in performance of motor tasks (all *P* > 0.05)
See and Treleaven (12)	None reported	Not stated	Reaction time, speed of movement, accuracy, co-ordination and tapping speed	Dominant and non-dominant limb	BEP_1_ for monitoring Human performance	1. Simple reaction time: time delay between a light stimulus and removing the hand from a central plate2. One-choice and 4-choice reaction time: participant to react by moving their hand to a target light stimulated touch plate from a central plate; choices of 1 or 4 target plates3. Movement speed – measured as distance from central plate to target plate/time for movement to occur.3. Tapping speed taps per second over time period of 10 s.4. Coordination participants alternatively tapping 2 narrow plates as fast and accurately as possible for 10 s; measured as accuracy (% correct hits) and average speed of movement	Significant difference in performance of motor task of 4-choice reaction time between WAD and healthy controls for both dominant (*P* = 0.04) and non-dominant hand (*P* = 0.02). All other sensorimotor tasks - no significant difference between WAD and controls

**Table 5:** Association between pain and self-rated function and upper limb sensorimotor task performance.

**References**	**Neck Pain** **Injury**	**Was the head** **neck moved** **as part of exposure** **and if so, to** **extreme range or not**	**Outcome Measures for** **pain, disability, function** **and mental status**	**Pain Scores**	**Is there a** **relationship between** **scores of disability/self-rated** **functioning and performance** **of sensorimotor task explored** **and if so, what** **were the results**	**Is there a** **relationship between pain** **and performance of sensorimotor** **task explored and** **if so, what were** **the results**	**Summary of upper** **limb S-M task errors**
Haavik and Murphy (20)	SCNP	Head moved to “almost end ROM”	Not reported	Not reported	N/A	N/A	Ab error control < SCNP group (P = 0.04)
Huysmans et al. (21)	Pain in neck and upper extremity	Head straight	Dutch version 30-item Disabilities of the arm, shoulder and hand questionnaire Perceived physical and mental exertion measured using the Borg-scale (0–10)	Duration of pain, 3.7 yrs (SD 2.8) (range 0.5–10). Worst pain in last 3 months 6 (3–10) Average pain in last 3 months 4 (2–9) Pain at day of measurement 4 (1–10).	Perceived physical exertion rated significantly higher for pain group than controls for both a small target and a large target. No significant difference between pain and control groups for perceived mental exertion	N/A	Position sense and acuity task: variable error significantly larger for participants with pain compared to healthy controls (*P* = 0.029); tracking task – larger mean distance between cursor and center of target (*P* = 0.038) pain group vs. control and larger standard deviation of distance from target (*P* = 0.008) pain group vs. control.
Knox et al. (13)	Chronic Whiplash (type II)	Yes, to just before participant reported increase in pain with movement	NDI, Speilberger State-Trait Anxiety Questionnaire (STAI)	Time since accident 22 (4–46) months; Mean NDI 42% (range 32–66) Baseline pain 2.0 (0–3.5) cm; Change in pain 0.9 (−0.1 to 2.2) cm during testing period	N/A	Significant correlation between baseline pain and Abs JPE (*r* = 0.46; *P* = 0.046) with neck in neutral position.Repeated measures ANOVA – greater baseline pain associated with greater abs JPE across all head-neck conditions (*P* = 0.03)	Changes in head-neck position increased absolute error of JPE in WAD group but not control (*P* = 0.048) with smaller angles of neck rotation. No difference in variable (*P* = 0.2) or constant (*P* = 0.8) error between WAD and healthy groups
Sandlund et al. (11)	Chronic whiplash II and III	Head straight	VAS pain, Pain Disability Index, 20 item Functional Self-Efficacy Scale, Short Form Health Survey SF-36	Minimum 6 months: Ranged from 6 months to 13 years (median 2.5)	Pearson's correlation used to explore association between position sense test outcome (VE) and questionnaire scores (i.e., were questionnaire scores predictors of VE). Moderate correlation between low self-rated physical functioning and low proprioceptive acuity (*P* = 0.042); High VE corresponded with low functioning The hypothesis that the degree of proprioceptive impairment is reflected by patients' symptoms and self-rated functioning was partially supported	No evidence of association between shoulder position sense and pain intensity (VAS and SG-36 scale)	WAD group showed significantly lower acuity in reproducing target compared to healthy controls (*P* = 0.003); as measured by VE
Sandlund et al. (22)	NS neck pain/ WAD	Head Straight	NDI, Short Form Health Survey - 36, VAS pain, Swedish validated version 27 Disability of the arm, shoulder and hand (DASH), TAMPA scale of Kinesiophobia (TSK), Self-efficacy Scale and Additional questions not covered by other questionnaires	Symptom duration NS: 60 (12–368) weeks; WAD: 73 (22–215) weeks;Mean (SD): VAS NS 47 ± 23; VAS WAD 60 ± 22	Strong association between end-point reaching acuity and neck function for both NS and WAD groups. For NS neck pain – neck ROM movements strongest predictors. For WAD, performing neck movements were strong predictors but also variables representing pain and limitation in performing activities involving lifting/carrying, poor balance and social functioning	Pain rating a significant predictor for VE in WAD group (*P* < 0.05) but not NS neck pain group	Neck pain, non-specific (*P* = 0.02) and WAD (*P* = 0.034) have significantly reduced end-point acuity in goal-directed reaching compared to control
Sittikraipong et al. (23)	Neck pain	Sitting, head position not stated but no active head movement exposure	Neck disability Index	VAS 4.6 ± 1.5; Neck pain duration (months) 24.6 ± 17	NDI scores moderately correlated with hand reaction time (*r* = 0.4, *P* = 0.005)	No correlations between reaction and response times and VAS/duration of neck pain	Reaction and response times significantly slower for pain groups compared to control (*p* < 0.001); hand-eye coordination task – neck pain group took longer to trace shape at most difficult level (*p* = 0.03)
Steinmetz and Jull, (24)	22 violinists with neck pain,	Presumably sitting, head position not stated but no active head movement exposure	Neck disability index; VAS Quantitative sensory testing: Thermal pain thresholds and pressure pain thresholds; both recorded over the cervical spine (C5-6)' PPT also recorded over tibialis anterior muscles	VAS 5 ± 2NDI (%) 18.6 ± 8.1Musicians with neck pain significantly reduced heat (*P* < 0.01) and increased cold pain thresholds (*P* < 0.01) compared to musicians without neck pain and healthy non-musicians.PPT: significantly lower threshold for musicians with neck pain compared to musicians without neck pain for both local (*P* = 0.02) and distal (*P* = 0.05) sites; No significant difference in PPT between musicians with neck pain and healthy controls	N/A	N/A	No significant difference between symptomatic and non-symptomatic musicians and non-musician group in performance of motor tasks (all *P* > 0.05)
See and Treleaven (12)	24 WAD	Sitting	Neck disability index (NDI %); Patient specific functional scale (average score out of 10 by totalling each activity/3); Disability of the arm, shoulder and hand (DASH/100)	VAS: 50.6 ± 20.1	Relationships between questionnaire score and BEP_1_ measures	High correlation between BEP_1_ (4-choice reaction time) and VAS pain scores	Significant difference in performance of motor task of 4-choice reaction time between WAD and healthy controls for both dominant (*P* = 0.04) and non-dominant hand (*P* = 0.02). All other sensorimotor tasks - no significant difference between WAD and controls

Finally, in order to investigate evidence in support of the role of pain and/or functional limitations in neck neuromusculoskeletal performance in contributing to an impairment in performance of upper limb sensorimotor tasks, additional data were extracted to determine whether there was a relationship between measures of disability or self-rated functioning and performance or pain and the results of sensorimotor tasks (see Tables 4, 5).

## Results

### Literature Search Results

**Figure 1:**
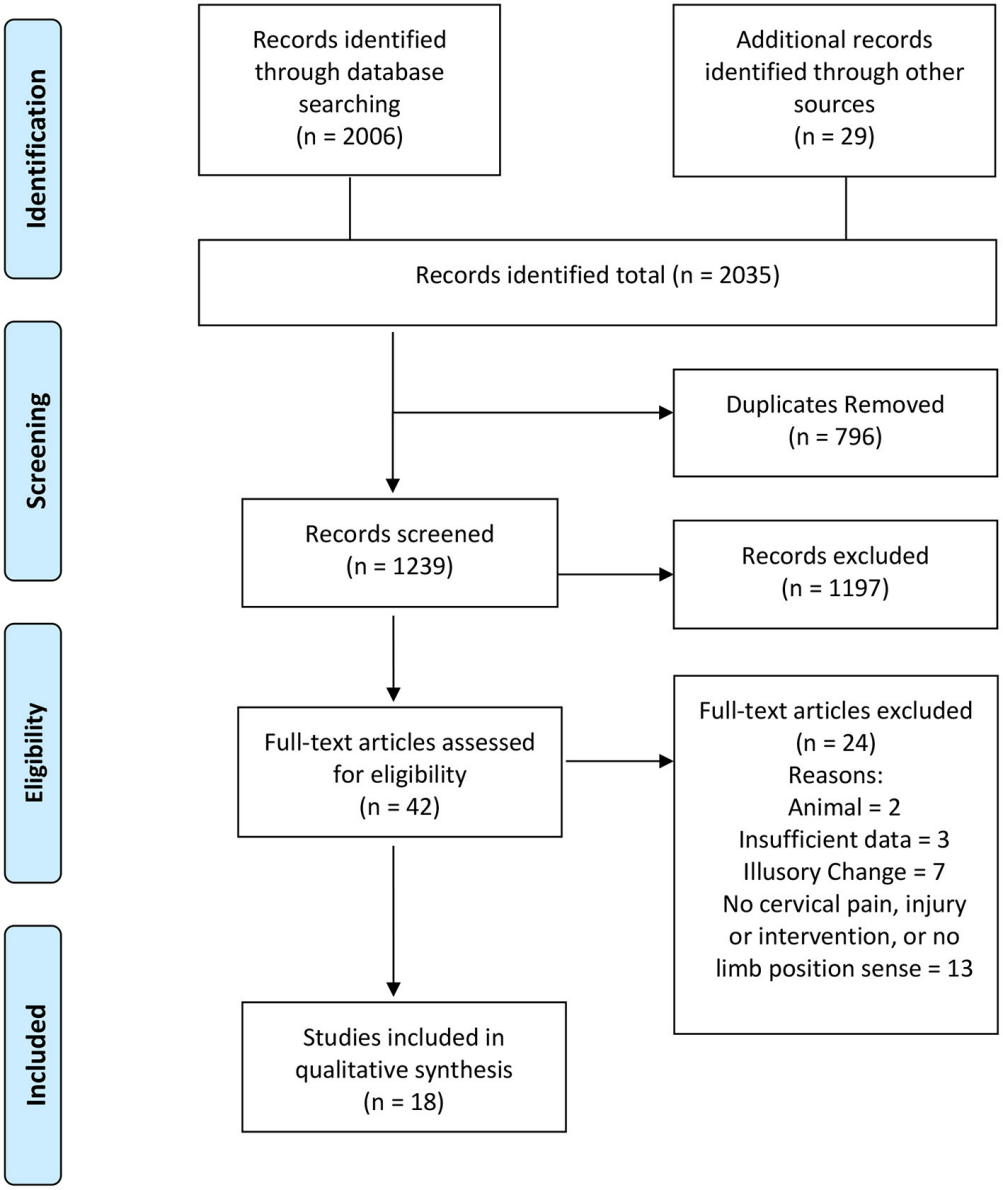
PRISMA flow diagram. Adapted from Moher et al. (17).

The online search strategy identified 2006 studies. Additional records identified through other sources numbered 29. Duplicates were removed leaving 1,239 studies. Of these, abstracts were screened by both reviewers based on the inclusion/exclusion criteria. From this process 42 studies were selected for full-text retrieval and assessed for eligibility. Consensus was reached to include 18 studies in the review. The most common reasons for rejecting articles were that they: were animal studies; didn't have sufficient detail; were not relevant (e.g., investigated head-trunk position sense without reference to the upper limb); or used artificial stimulation (e.g., vibration or galvanic stimulation). Search results and the selection process are summarized in Figure 1. Heterogeneity in designs of studies prevented pooling of data, so qualitative analysis was undertaken.

From the studies included in the review, all were prospective. Eight were cross-sectional studies and ten were case series. The cross-sectional studies compared healthy participants and participants with neck pain and/or injury (11–13, 20–24); and the case series assessed a healthy cohort in pre-test/post-test studies (14, 25–33).

### Participant Demographics and Health Status

Participant demographics and health status were described in appropriate detail in 17/18 (94%) studies and included: the inclusion/exclusion criteria; the number of participants; participant gender, age and health condition; and questionnaires used to evaluate pain, disability, function and mental status in clinical cohorts (see Table 2) (11–14, 20–27, 29–33). Of the eight cross-sectional studies, participant groups included healthy 8/8 (100%), subclinical neck pain (1/8) (13%) (20), neck pain 4/8 (50%) (21–24) and those suffering WAD in 4/8 50%) (11–13, 22) studies. Three of four of the WAD studies (11, 13, 22) graded the severity of WAD as II or III, in accordance with the Quebec Task Force classification on whiplash-associated disorders (34). This classification is a widely accepted system for describing the different levels of dysfunction and symptomatology for whiplash. Participants for the majority of studies were predominantly aged 18–45 years (15/18, 83 %) (11, 13, 14, 20, 22–27, 29–33) with 3/18 (20%) including participants who were over 45 years old (12, 21, 28). Information was also presented on gender in 15/18 studies (83%) (11–13, 20–25, 28–33), handedness was reported in 14/18 (78%) (11, 13, 20–22, 25–33) studies and dominant upper limb reported as used in performance of upper limb kinesthetic tasks by all participants in 13/18 studies (72%) (11, 21–23, 25–33). One study assessed motor performance of dominant and non-dominant hands (12).

A high level of detail of inclusion/exclusion criteria was used to recruit participants and was reported in 13/18 (72%) studies (11–14, 20–24, 29–32). Questionnaires were used to measure characteristics of participants including pain, disability, function and/or mental status in relevant studies (see below).

In those studies that included participants experiencing pain, outcomes of pain duration and intensity were recorded in 6/8 (75%) (11–13, 21–23) and 6/8 (75%) (12, 13, 21–24) studies, respectively. Outcome measures used to evaluate the various dimensions of neck pain/injury included the Neck Disability Index questionnaire and pain visual analog scales used in 7/8 (88%) studies (11–13, 21–24) and the Quality of life, self-efficacy, Disability arm/shoulder/hand and TAMPA scale of kinesiophobia questionnaires variously used in 5/8 (63%) studies (11–13, 21, 22). Out of the remaining 10/18 (56%) studies which used a healthy cohort only, one study (31) confirmed the absence of neck pain using the Neck Disability Index.

### Risk of Bias and Measures of Outcomes

The risk of bias analysis for all studies included in this review is presented in Table 1.

The overall quality of all studies was moderate to high with 17/18 (94%) (11–14, 21–33) studies having believable results as indicated by Viswanathan and Berkman (18). Fifteen of the 18 studies (83%) (11–14, 21–25, 27, 29–33) achieved a score of 4/6 or greater resulting in a quality of score of high. The remaining studies scored more than 2/6 (20, 26, 28) indicating a quality score of moderate.

All studies were prospective, used objective measures and had a high level of detail in describing the exposure used. Inclusion/exclusion criteria were clearly stated in 13/18 (72%) studies (see above), with 11/18 (61%) measuring inclusion/exclusion criteria using objective measures (11–14, 21–24, 30–32) and all used appropriate statistical methods. One study did not accurately report statistical analysis (20).

### Definitions of Clumsiness

Details of definitions of clumsiness, and/or investigations of the effect of head-neck position and/or pain on upper limb kinesthesia are shown in Table 3. Clumsiness was defined in 3/18 (17%) studies (11–13) where each reported it as a clinical manifestation associated with neck pain and/or injury. Two studies operationally defined this as a deficit in coordination of upper limb movement or an impairment in upper limb proprioception (11, 13). Both Sandlund et al. (11) and Knox et al. (13) made reference to Bring and Westman (7) who reported fumbling as a clinical sign/symptom of whiplash. Knox et al. (13) used a cross-sectional study (WAD and healthy participants) to examine accuracy of reproducing a target elbow angle after head and neck movement to a point before pain was experienced. The change in head-neck position was, therefore, less than full range of neck motion. The aim of their study was to investigate the effect of changes in head and neck position on the perception of elbow position in people with chronic and disabling neck pain after a whiplash injury. The group suffering WAD recorded reduced proprioceptive acuity compared to their healthy counterparts when the head-neck of the healthy group was moved to the same average degree of rotation as the WAD group away from midline (neutral) position. Sandlund et al. (11) also used a cross-sectional study design (WAD and healthy groups) but examined shoulder joint movement to previously defined target positions with no change in head position. They hypothesized that people with WAD have impaired shoulder proprioception.

Clumsiness was not explicitly referred to in the remaining studies. However, all studies made reference to the role of the neck in interpreting the position of body segments relative to each other and/or in extra-personal space.

### Associations Between the Neck and Changes in Accuracy in Completion of Upper Limb Sensorimotor Tasks

The position/s and/or movement/s of the neck and the form of the upper limb task are detailed in Table 4. In particular this table identified: the head-neck movement performed; the position of the body during upper limb task performance; the upper limb kinesthetic task performed, including the visual condition of the participant during the performance of the upper limb task; the instruments used for measurement of upper limb performance; the outcome measures reported; and the results of the studies.

Ten of 18 (59%) studies investigated a change in head position on upper limb task performance with 6/10 (60%) of these studies passively changing head position (13, 14, 20, 25, 29, 32). One study (27) induced neck rotation by passive movement of the trunk against a fixed head while neck muscles were relaxed or when neck muscles were actively contracted and a further two studies (28, 33) required participants to actively move their head-neck to different angles of rotation. Fookson et al. (28) stated that the neck was moved to its end ROM while participants of the Rosetti et al. (33) study were required to move their head-neck incrementally to 80 degrees. Berger et al. (26) did not indicate whether a change in head-trunk position was achieved actively or passively. Six of 18 (33%) studies maintained the head in a straight-ahead position with each study investigating participants with neck pain and/or those having a WAD (11, 12, 21–24). Two studies induced dorsal neck muscle fatigue prior to measurement of the upper limb kinesthetic task (30, 31).

Studies investigated the role of neck pain/injury, or changes in head-trunk position, or muscle fatigue on: (i) an active upper limb goal directed movement such as the participant pointing at a target 7/18 (39%) (21, 22, 26–28, 31, 33); (ii) upper limb joint position sense (JPS) 5/18 studies (28%) (11, 13, 14, 20, 30); (iii) drawing tasks 3/18 (17%) (25, 29, 32); (iv) reaction and response times 3/18 (17%) (12, 23, 24) and (v) upper limb tracking task 3/18 (17%) (21, 23, 31). Experimental protocol descriptions for upper limb tasks and head-neck position/s or movement/s were detailed in all but one study (26) as was the visual condition of participants 18/18 (100%). The test position of the participant was stated in the majority of studies, 6/18 (33%) supine (13, 14, 20, 25, 29, 32), 2/18 (11%) (21, 30) standing and 9/18 (50%) seated (11, 21, 22, 25–28, 31, 33). Three studies did not explicitly indicate participants' posture during testing although it may be presumed that participants were seated (12, 23, 24).

All studies used objective means of measurement for upper limb task performance. The types of instrumentation used included electromagnetic trackers used in 6/18 (33%) studies (11, 22, 25, 27, 29, 32); an electrogoniometer used in 3/18 (17%) studies (13, 14, 20); Infra-Red LEDs (IR-LEDs) used in 4/18 (22%) studies (26, 28, 30, 33) and a digitized tablet or touchscreen used in 3/18 (17%) studies (21, 23, 31). One study (23) measured response and reaction time using a hand-held electronic timer with modified computer mouse and two studies used the Human Performance Measurement/Basic Elements of Performance (HPM/BEP) device (12, 24).

### Association Between Neck Pain, Natural Interventions Applied to the Neck and Performance of Upper Limb Sensorimotor Tasks

Only eight of the 18 studies (44%) included in this review involved a clinical cohort where participants suffered a neck complaint. These complaints included subclinical neck pain (20), chronic neck pain (21–24) and WAD (11–13, 22). All studies (100%) used various forms of an upper limb sensorimotor task (that is a task involving a sensory stimulus and an active upper limb movement in response to the sensory stimulus). Two of the eight studies (25%) involved a passive head movement prior to assessment of task performance (13, 20). Of these, one involved movement of the head and neck “almost to the end of their range of motion” (20) while the other passively moved the head and neck to a point that avoided increasing each participant's pain or discomfort (13). In three studies (38%), the head-neck remained in a head-neck straight ahead position (11, 21, 22) while a further three studies (12, 23, 24) (38%) did not indicate head position although given the type of task performed, it would be expected that the head was maintained in a neutral position. All but one of the cross-sectional studies (88%) demonstrated a deterioration in performance of the sensorimotor task when compared to a healthy control group. The study not reporting a reduction in performance of an upper limb sensorimotor task was conducted with a cohort of professional violinists/violists (24).

Ten of the 18 studies (56%) investigated a healthy cohort only. The majority of these studies, 7/10 (70%) involved passive head-neck or neck-trunk movement approximating the extreme of head neck range of motion (14, 25–28, 32, 33). Two of ten studies (20%) induced dorsal neck muscle fatigue (30, 31), while one study used the return phenomenon which consisted of passively tilting the head-neck to one side and maintaining that position for 15 min prior to the head-neck being returned to the head straight ahead position (29). In all studies, these natural maneuvers resulted in a deterioration in performance of upper limb sensorimotor tasks.

### The Relationship Between Pain, Disability and Self-Rated Functioning in Contributing to an Impairment in Performance of Upper Limb Sensorimotor Tasks

Of the eight studies included in this review that conducted prospective, cross-sectional research all broadly included a neck pain group and a healthy group as previously reported. We were interested in exploring whether any relationship existed between upper limb sensorimotor task performance and measures of pain or self-rated function and disability. The results of this analysis are provided in Table 5. Six of the eight studies specifically reported on this relationship (11–13, 21–23). Of these, 3/6 studies reported a significant association between measures of pain and upper limb sensorimotor task performance (12, 13, 22); that is, the more intense the pain, the more impaired the upper limb task performance. Four of six studies reported an association between disability, self-rated functioning and sensorimotor task performance (11, 21–23) and a similar relationship applied to the intensity of pain. Further, 3/6 of these studies reported an increased variability of errors in task performance (11, 21, 22).

## Discussion

This systematic review found a small body of literature of moderate to high quality, with all but one study, demonstrating that in the presence of neck pain or injury or when a natural intervention is applied to the head-neck that provokes the neck to function close to extreme limits in a healthy cohort, this is associated with a deterioration in the accuracy of performance of upper limb sensorimotor tasks, or the accurate perception of upper limb joint position. As far as we are aware, this is the first study to link experimental and clinical studies that demonstrate disordered sensorimotor integration to altered neck sensory input. While only three studies explicitly referred to clumsiness/fumbling (11–13) and two of these referred to this clinical phenomenon as a deficit in coordination of upper limb movement or impairment of upper limb proprioception (11, 13), all studies except one in this review arrived at a consistent result. The result of this review highlights the importance to clinicians to specifically investigate whether their patient experiences clumsiness or fumbling associated with their neck pain or injury and to implement rehabilitative strategies to address this issue.

This review also uncovered a pattern of evidence that supports the proposal that pain itself, or decreased levels of self-rated functioning in the presence of neck pain/or injury is associated with a decrease in the performance of various upper limb sensorimotor tasks.

### Quality of Studies

As noted, analysis of the details of these studies was restricted to a qualitative analysis principally due to the variability in study designs as well as outcome measures used to measure upper limb sensorimotor task performance. Nevertheless, the overall quality of the studies was considered moderate to high. We conclude that the strong consistency of the results of this review overrides any deficiency in the assessment of the quality of the studies specifically as this relates to the reporting of inclusion and exclusion criteria, the reporting of statistical analyses or some lack of detail in describing exposures.

### Measures of Upper Limb Sensorimotor Performance

All the studies included in this review can be broadly divided into one of two categories. The first category (5/18 studies) includes those studies that examined the performance of a sensorimotor task in intra-personal space in the presence of neck pain only (20), neck pain associated with injury (11, 13) or in a healthy cohort when a natural intervention was applied (14, 30). That is, sensorimotor tasks involved on-going assessment of segment-to-segment movement and position. The second category included studies (13/18) where sensorimotor performance involved the assessment of the position and/or movement of an object in extra-personal space as well as knowledge of the position and movement of the upper limb (12, 21–29, 31–33). In the first category the conclusion reached by all studies was that changes in head-to-trunk orientation, extensor neck muscle fatigue or in the presence of neck pain or injury is associated with a decrease in accuracy in estimating a previously presented upper limb joint position. This inaccuracy was interpreted as a disruption in the updating of the internal body schema as a consequence of altered neck afferent input.

The second category of studies is more complex to analyze because these studies examined upper limb task performance on multiple levels. On the first level, the CNS must identify the position and movement of the upper limb, that is, it makes reference to an internal representation of the body as described above. The CNS must do this while, on the next level, the upper limb moves and positions itself in relation to an object in external space. Therefore, the CNS must also encode an external target position. Blouin et al. (27) concluded that this is achieved by the CNS using a trunk-centered reference system allowing the external target to be located relative to changing body position. This is achieved partly by using proprioceptive input from the neck and integrating this information with that of vision although there is evidence that proprioception may play a dominant sensory input in motor planning (32). Indeed as articulated by Guerraz et al. (32), regardless of the sensorimotor task, changes in the positional relationship between the head-trunk, results in an internal bias in the representation of the head-trunk which is used to accurately navigate the position and movement of the self's body segments to the location of the external target.

In this regard it is worth noting the study of Guerraz et al. (29) who used the “return phenomenon” as the intervention to change head-on-neck orientation. The return phenomenon occurs as a result of a change in the *perception* of position of the head-to-trunk position rather than the actual head-to-trunk position, the perception of which changes over time. This was first demonstrated by Gurfinkel and Levik [as cited by Levik (35)]. In the Guerraz et al. study (29) participants were asked to repeatedly draw a line “aligned with their trunk.” The magnitude of angular deviation of the line drawing task gradually diminished during head tilt, but never entirely. When the head was returned to be aligned with the trunk, participants reported the perception that their head tilted in the opposite direction with respect to the initial direction of tilt. Interestingly so too did the angular deviation of the drawn straight line with the degree of deviation decreasing as the perception of head tilt reduced.

Guerraz et al. (29) argued that both the position of the head during the initial stages of head tilt and the resultant deviation in line drawing represented a bias in the trunk-centered reference system of the internal body schema provided by neck proprioception alone. Over time, as participants perceived that their head slowly returned to a neutral position so too did the extent of line orientation of the drawing task although there was no significant correlation between them. The authors argued that the return phenomenon was responsible for participants' perception of their head position rather than the actual head position and this was reflected in the motor task of line drawing. This study highlighted the depth of the concept of the system of internal representations of body constructs both within intrapersonal space as well as the body's relationship to external objects in the environment as described by Levik (35). Therefore, the results of the study during the initial stages of head tilt are consistent with the finding that neck proprioceptors contribute to the continual updating of the internal body schema.

In summary, all the studies in this second category, except one (24), arrived at the same conclusion, that, there is a deterioration in the performance of sensorimotor tasks in the presence of neck pain, and/or an injury to the neck or when the head-neck functions close to the endpoint of its range.

### Is There a Relationship Between Neck Pain/Injury and Measures of Clumsiness?

Eight of the 18 studies of this review investigated upper limb sensorimotor task performance in the presence of neck pain. All but one of these studies determined that in the presence of neck pain, the accuracy in performance of sensorimotor tasks was reduced. Furthermore, six of these studies reported a positive association between the severity of reduction in performance of the upper limb task and levels of pain, physical functioning or levels of reported self-efficacy. While the number of studies that reported these findings is small and the variety of testing procedures varied, the results of this review point to a relationship between neck pain where an individual experiences a higher level of reduced physical functioning or self-efficacy and clumsiness.

It is of interest to note here the findings of (24) the only study that did not report the association. Their study investigated sensorimotor function in violin and viola players with and without neck pain. They found no difference in the performance of motor tasks between violinists and violists with and without neck pain. As concluded by these authors it may be that fine motor tasks associated with the playing of their instruments may be a more appropriate way to assess whether a deterioration in function is actually occurring. Further, to our knowledge, it is not yet established whether the types of finely developed motor skills of these musicians might mask the effects of a deterioration in sensorimotor function as measured by standard upper limb sensorimotor tests.

The results of our review also demonstrate that when the neck is required to function near the limits of its normal range, that here too, a deterioration in the performance of upper limb sensorimotor tasks does occur. All in all, the results of this review provide strong evidence that disturbances in and of the neck are associated with clumsiness.

### The Neck, the Internal Body Schema and the Clinical Symptom of Clumsiness

How do we know the position of our limb in relation to the rest of our body or where it has moved if we cannot see it? How do we perceive where our different body segments are positioned in relation to the external world? Scientists refer to this knowing as the “body schema” where the brain maps and continually updates the body's “shape and posture” (36). It is well-known that the concept of the internal body schema is reliant on the collective proprioceptive inputs to both construct and update the internal body schema (37) including those of the neck (16). It is also well-known that neck muscles have a high density of proprioceptors, notably muscle spindles (38–40) and that projections from these receptors enter the CNS reaching multiple areas of the brain including the vestibular system - an important integration center for sensorimotor control (8, 16). Proprioceptive information also reaches the cerebral cortex and “tunes motor commands for spatially oriented movements” as stated by Palliard (41).

In a recent review by Pettorossi and Schiepatti (16), they reported there is strong evidence that proprioception from neck muscles is significantly involved in the construction and updating of the position and movement of the body and its segments in order to inform reference frames for movement. Haavik et al. too (42), identified that the function of the deep (small) paraspinal muscles and their proprioceptive input are capable of altering (or maintaining) an accurate record of the brain's internal body schema.

Therefore, it seems reasonable that given the central role of neck proprioceptors in the construction and updating of the internal body schema that, in the face of a disturbance in neck function such as occurs in the presence of pain or injury, there is the potential to disrupt the transmission of proprioceptive signals or to distort the processing of proprioceptive input which, in turn, alters the internal body schema.

This review also provided support for the contribution of pain in disrupting the body schema. While the studies included in this review did not use quantitative sensory testing of pain to examine the presence of central sensitization except for the article of Steinmetz and Jull (24), we did find evidence for an association between the extent of deterioration in performance of upper limb sensorimotor tasks and self-reported measures of pain, physical functioning and levels of reported self- efficacy. There is evidence, that central sensitization is responsible for ongoing reports of pain and disability in people suffering chronic WAD (43) and altered central pain processing in those with non-traumatic neck pain (44). Future studies need to examine if disturbed body schema is part of central sensitization, and partly responsible for deteriorated quality of life and disability associated with chronic musculoskeletal pain.

### Strengths and Weaknesses

This is the first review that has attempted to systematically investigate whether there is an association between clumsiness and neck pain injury. Strengths of this study include that it followed the Cochrane protocol (17) with a comprehensive search conducted among key databases and independent reviewers selecting studies and extracting the data.

Overall, the quality of evidence was moderate to high; and importantly, the results, regardless of whether studies involved a clinical sample or healthy volunteers, consistently showed that when the neck's neuromusculoskeletal elements were compromised due to pain [cf. (24)], injury, fatigue or extreme range of movement, that this was associated with increased errors in upper limb performance. Another strength of this review is that the authors came from both neuroscience and clinical disciplines. The experience from these different backgrounds allows for dual perspectives to ensure the findings of neurophysiological studies remained clinically relevant.

The main weakness of this review is the relatively low number of studies found, 18 in total, with only three studies (11–13) describing clumsiness as a clinical manifestation associated with neck pain and injury and one (13) operationally defining clumsiness. This issue was identified during our initial search and addressed by developing a range of terms to represent clumsiness to broaden the literature search. In addition, the sample size of each study was small, ranging from 6 to 120 participants. The outcome measures applied were also diverse. All together it was not possible to pool all data to conduct a meta-analysis. Nevertheless, all studies provided sufficient details of their sample, interventions and outcomes used to measure performance of upper limb sensorimotor tasks to enable us to conduct a qualitative review and assess the association between the neck and upper limb performance.

### Clinical Implications

Given the findings of this review the major question that arises is what do we know about current prevalence of clumsiness in patients who present to our clinics? We were able to identify only one study from 2003 (5) reporting 30% of their sample (*N* = 102) of people suffering persistent WAD with an accompanying symptom of dizziness (*N* = 76) who also complained of clumsiness. Yet, the results of this review indicate that disturbances in upper limb sensorimotor task performance might be expected to occur more frequently in clinical populations presenting with conditions other than WAD given that a deterioration in performance of upper limb tasks can occur even when the neck is required to function at more extreme limits. Examples of such neck function may include the operation of machinery that requires the head-neck to be turned to near end range of motion, or fatigue of neck muscles when the head-neck is required to assume a static posture for a prolonged period.

A further area of much needed research is in older people with neck pain. Vogt et al. (45) found a prevalence of between 11 and 19% (male/female) musculoskeletal pain involving the neck and shoulder in 70–79 year-old people living in the community in a study that collected data between 1997 and 1998. A more recent study by Quek et al. (46) found higher levels of dizziness handicap in older people with neck pain when compared to those without neck pain. This latter study used the dizziness handicap inventory as part of a battery of tests to examine characteristics of older people with neck pain who also presented with impaired postural stability. These authors concluded that neck-pain induced deficits in indices of postural instability including dizziness may have occurred as a result of deficits in neck proprioception as a consequence of neck pain. It is well-established that dizziness in older people is highly prevalent and that prevalence increases with age, while causes of dizziness remain unknown in 20–40% of patients reporting to medical practice physicians according to Dros et al. (47). Given the finding of Treleaven (5) that a substantial proportion of WAD sufferers also described symptoms of clumsiness, there is a need for further investigation of clumsiness and neck pain in older people to enable non-pharmacological treatment and rehabilitation strategies for this age group.

We contend that there is a need to specifically address the clinical issue of clumsiness including investigations of clumsiness in older people and in the workplace, the latter to discover whether tasks that require the head-neck to function at extreme limits are associated with a deterioration in upper limb sensorimotor task performance.

## Conclusion

This review found limited research reporting on the specific symptom of clumsiness despite reports of a prevalence of 30% of WAD sufferers reporting symptoms of clumsiness as a consequence of neck injury (5). Disturbances in neck sensory input in both healthy people and those with neck pain and/or injury are associated with reductions in the acuity of upper limb kinesthetic sense and a deterioration in sensorimotor performance. Further research should investigate the specific role that a disruption in neck muscle proprioceptive input plays in alterations to the internal body schema in both healthy people and those with musculoskeletal conditions. This will enable the development of evidence-based manual therapy treatment and rehabilitation strategies for restoring the distorted body schema associated with neck pain/injury.

## Data Availability Statement

The raw data supporting the conclusions of this article will be made available by the authors, without undue reservation.

## Author Contributions

SH: concept development, study selection, literature search, data extraction, data analysis, data interpretation, and manuscript draft. ZZ: concept development, study selection, data analysis, data interpretation, manuscript draft, and manuscript revision. JK: literature search, data interpretation, and manuscript draft. DV: concept development, data interpretation, and manuscript draft. BP: concept development, study selection, literature search, data extraction, data analysis, data interpretation, manuscript draft, and manuscript revision. All authors contributed to the article and approved the submitted version.

## Funding

SH was supported by a scholarship from the Australian Chiropractors' Association and an RMIT University postgraduate scholarship.

## Conflict of Interest

The authors declare that the research was conducted in the absence of any commercial or financial relationships that could be construed as a potential conflict of interest.

## Publisher's Note

All claims expressed in this article are solely those of the authors and do not necessarily represent those of their affiliated organizations, or those of the publisher, the editors and the reviewers. Any product that may be evaluated in this article, or claim that may be made by its manufacturer, is not guaranteed or endorsed by the publisher.
