# Supplementary material for: Does My Neck Make Me Clumsy? A Systematic Review of Clinical and Neurophysiological Studies in Humans
Source: Front Pain Res (Lausanne). 2021 Oct 11;2:756771. doi: 10.3389/fpain.2021.756771 (PMC8915649; doi:10.3389/fpain.2021.756771)
Supplement: Supplementary file 1 [file Data_Sheet_1.zip › 756771_Polus_Supplement_B.docx]

**Supplement B: PubMed Database Search Terms**

| Search Number | Search Terms |
| --- | --- |
| #1 | Search “Neck injuries”[MeSH Terms] OR neck injuries[Text Word] OR "neck pain"[MeSH Terms] OR neck pain[Text Word] OR Whiplash OR “Whiplash  Associated Disorder*” OR Healthy OR cervical |
| #2 | Search Clumsy OR Clumsiness OR Proprioception OR Fumbling OR Coordination OR "ataxia"[MeSH Terms] OR ataxia[Text Word] OR "kinesthesis"[MeSH Terms] OR kinesthesis[Text Word] OR "psychomotor performance"[MeSH Terms] OR psychomotor performance[Text Word] OR Dystaxia OR "dyskinesias"[MeSH Terms] OR dyskinesias[Text Word] |
| #3 | Search “Joint Position Error” OR “Joint Position Sense” OR Electrogoniometer  OR “Electromagnetic tracking” OR Goniometer OR “Position Sense” |
| #4 | Search "upper extremity"[MeSH Terms] OR upper extremity[Text Word] OR "lower  extremity"[MeSH Terms] OR lower extremity[Text Word] OR elbow OR shoulder OR wrist Or arm OR hand OR axilla OR leg OR Knee OR ankle OR foot OR hip OR  “lower limb” |
| #5 | #1 AND #2 AND #3 AND #4 |
